# The Nanoworld of the Tripartite Synapse: Insights from Super-Resolution Microscopy

**DOI:** 10.3389/fncel.2017.00374

**Published:** 2017-11-24

**Authors:** Janosch P. Heller, Dmitri A. Rusakov

**Affiliations:** ^1^UCL Institute of Neurology, University College London, London, United Kingdom; ^2^Institute of Neuroscience, University of Nizhny Novgorod, Nizhny Novgorod, Russia

**Keywords:** synapses, astroglia, tripartite synapse, super-resolution microscopy, plasticity

## Abstract

Synaptic connections between individual nerve cells are fundamental to the process of information transfer and storage in the brain. Over the past decades a third key partner of the synaptic machinery has been unveiled: ultrathin processes of electrically passive astroglia which often surround pre- and postsynaptic structures. The recent advent of super-resolution (SR) microscopy has begun to uncover the dynamic nanoworld of synapses and their astroglial environment. Here we overview and discuss the current progress in our understanding of the synaptic nanoenvironment, as gleaned from the imaging methods that go beyond the diffraction limit of conventional light microscopy. We argue that such methods are essential to achieve a new level of comprehension pertinent to the principles of signal integration in the brain.

## Introduction

Information processing in the brain relies on synaptic connections between nerve cells. It has been, however, somewhat a revelation that the process of signal formation and handling by neural circuits also involves an intimate relationship between neurons and nearby astroglia. Initially, astrocytes were thought of as exclusively structure-supporting cells. Later, two key roles of astroglia in signal transmission have emerged, rapid uptake of neurotransmitters such as glutamate (Bergles and Jahr, 1997; Diamond, 2001), and extracellular potassium buffering (Hertz, 1965; Orkand et al., 1966). Finally, the discovery of Ca^2+^ waves within and among astrocytes (Cornell-Bell et al., 1990; Nedergaard, 1994; Parpura et al., 1994) has prompted the concept of astroglial excitability, hence the ability to integrate and transfer physiological signals.

Most astrocytes feature sponge-like morphology *in vivo*, with thousands of fine protrusions filling up the extracellular space, whereas individual cells occupy adjacent, non-overlapping tissue territories (Bushong et al., 2004; Halassa et al., 2007). At many excitatory synapses in the brain, fine perisynaptic astrocytic processes (PAPs) are in close contact with pre- or postsynaptic specializations. It has been found that PAPs and synapses are in a continued exchange of molecular signals regulating synaptic transmission and its use-dependent plasticity (for recent reviews see Hamilton and Attwell, 2010; Araque et al., 2014; Rusakov et al., 2014; Bazargani and Attwell, 2016). This exchange has prompted the notion of the tripartite synapse (Haydon, 2001), acknowledging astroglia as an important third partner in signal transfer between presynaptic and postsynaptic cells. More recently, this terminology has been extended to the “tetrapartite synapse” which includes the extracellular matrix (Dityatev and Rusakov, 2011) or the “quad-partite synapse” which incorporates microglial processes (Schafer et al., 2013).

The intricate interactions between synapses and astrocytes have been difficult to visualize using conventional microscopy because most astroglial compartments are smaller than the diffraction limit of light. The advance of electron microscopy (EM), and in particular when equipped with three-dimensional (3D) reconstruction from serial sections, has revealed the tripartite synapse structure in some intricate detail (Špaček, 1985; Ventura and Harris, 1999; Lehre and Rusakov, 2002; Witcher et al., 2010; Patrushev et al., 2013). Subsequent advances in immuno-EM have uncovered a plethora of signaling molecules that are located in PAPs (reviewed in Heller and Rusakov, 2015; Bazargani and Attwell, 2016). Moreover, EM studies have indicated that PAPs preferably approach thin spines rather than large ones (Medvedev et al., 2014). More recently, high-throughput 3D EM methods, such as serial blockface-scanning or focused ion beam scanning EM, have further moved the goalposts of synaptic 3D reconstruction studies, by increasing considerably (z-stack) sectioning resolution, and thus improving quantitative description of synaptic elements, compared to traditional serial-section TEM (Blazquez-Llorca et al., 2015; Bosch et al., 2015).

One important limitation of EM studies, however, is that they can only be carried out in fixed tissue preparations, thus providing correlational comparisons between different samples. In turn, existing immuno-EM methods have not been able to provide a contiguous 3D representation of the (immunogold-labeled) signaling proteins expressed in PAPs. Nonetheless, EM studies have discovered that the astroglial coverage of synapses may depend on the physiological state, local neuronal activity, induction of synaptic plasticity, or certain behaviors (Wenzel et al., 1991; Jones and Greenough, 1996; Oliet et al., 2001; Lushnikova et al., 2009; Bernardinelli et al., 2014; Ostroff et al., 2014). Subsequent studies in live tissue that employed confocal and two-photon excitation microscopy have indicated that the spatial relationship between PAPs and the adjacent synapses is indeed dynamic (Hirrlinger et al., 2004; Haber et al., 2006; Bernardinelli et al., 2014; Perez-Alvarez et al., 2014), light diffraction being the limiting factor.

More recently, the successful implementation of fluorescence recovery after photobleaching (FRAP) and single-particle tracking (SPT) techniques paved the way for monitoring dynamic changes in nanoscopic cellular compartments at super-resolution (SR), i.e., beyond the diffraction limit of conventional optical microscopy. In brief, the time course of FRAP depends on the diffusion rate (and the proportion of immobile fraction) for the fluorescent label of interest (Axelrod et al., 1976). In contrast, SPT methods rely on the labeling of individual molecules in live cells using bright fluorescent nanoparticles, such as quantum dots. The nanoscale localization and tracking of labeled molecules then proceeds through the registration of intermittent quantum dot “blinking” followed by the deconvolution of the signal point-spread function (PSF; reviewed in Choquet and Triller, 2003). Stimulated-emission depletion (STED) microscopy uses a second excitation light channel in which light forms a doughnut-shaped PSF to damp (deplete) excitation at the periphery of the first channel PSF: this narrows the emission spots enhancing resolution beyond the diffraction limit (Klar et al., 2000). In structured illumination microscopy (SIM), bar code-like excitation patterns are shifted and rotated in the excitation path, creating high-frequency information, which can be extracted using specialized algorithms to reconstruct a super-resolved image (Gustafsson, 2000; Gustafsson et al., 2008; Schermelleh et al., 2008). Single molecule localization microscopy (SMLM), which encompasses two generic approaches (and their specific applications), photo-activated localization microscopy (PALM) and stochastic optical reconstruction microscopy (STORM), is based on localizing the point source of fluorescence (which can be one molecule) from its detected image (Betzig et al., 2006; Rust et al., 2006; Fölling et al., 2008). Similar to the SPT technique, the exact position of the fluorescent label is reconstructed through repeated stochastic excitation of a small, sparsely distributed fraction of fluorescent molecules per imaging cycle and the subsequent PSF deconvolution.

Each of the SR techniques has certain advantages and shortcomings. For instance, resolution in STED microscopy depends directly on the intensity of the excitation and depletion laser light, which if excessive might produce phototoxic effects. Nonetheless, high imaging speed and advanced optical sectioning makes STED imaging applications feasible in live tissue. Another advantage of the technique is the applicability of common fluorescent proteins and dyes.

SIM is another highly flexible SR imaging method. It does not require specific fluorophores, and it allows for multi-color imaging in living cells and organisms. Importantly, it does not require high intensity excitation light thus carrying relatively low risk of photo-damage. However, maximal resolution of SIM is about three times lower than that of STED or SMLM.

STORM imaging has mainly been used in fixed cells and tissue, and it relies on labeling of endogenous or overexpressed proteins equipped with antibody constructs. Therefore, it requires good tissue penetration for the antibodies and dyes. Even though STORM enables multi-color imaging, it is only specific dye combinations that could work in certain buffer formulations.

In general, SMLM methods provide the greatest enhancement of resolution, which is effectively limited only by the size of the fluorophore molecule and the opto-mechanical stability of the system. Importantly, not only can various SR techniques be used in living cells (Jones et al., 2011; Owen et al., 2012; Bethge et al., 2013), they could also document the dynamics of molecular movement. One type of SMLM (sptPALM) uses the expression of a photoactivatable fluorescent protein fused to the protein of interest, thus enabling reconstruction of hundreds of molecular trajectories (Manley et al., 2008; Rossier et al., 2012). Another method, universal point accumulation for imaging in nanoscale topography (uPAINT), relies on the addition of fluorophores during the imaging process, which provides the dynamic imaging of continuously labeled, arbitrary membrane biomolecules in living cells (Giannone et al., 2010). In contrast to the nanoparticle-based SPT techniques, in which a few molecules are followed for prolonged periods of time, sptPALM and uPAINT image single-molecule trajectories at very high densities, for short periods of time (Cognet et al., 2014; Sibarita, 2014). A detailed description of these and related techniques could be found in some excellent recent reviews (Schermelleh et al., 2010; Sydor et al., 2015; Turkowyd et al., 2016; Minoshima and Kikuchi, 2017).

Our aim here is to overview and discusses some notable recent findings that took advantage of SR microscopy in visualizing synaptic and astrocytic structures in mammalian cells. We refer the reader elsewhere for the description of some important advances made in the model organism *Drosophila* (Liu et al., 2011; Ehmann et al., 2014; Bosch et al., 2015; Böhme et al., 2016; Bademosi et al., 2017). Similarly, in-depth reviews focusing on the synaptic receptor machinery, as revealed with SR microscopy, could be found in the recent literature (Ribrault et al., 2011; Czöndör et al., 2012; Choquet and Triller, 2013; Compans et al., 2016). Our focus here will be on the progress in our understanding of the relationship between molecular machineries acting at the presynaptic terminal, the postsynaptic dendritic spine, and the perisynaptic astroglia—as being discovered with SR methods. The rationale behind this approach is to bring together and illustrate SR-generated data coming from inter-disciplinary research groups that do not always communicate among themselves and have not always been directly related to synaptic physiology research.

## SR Imaging of the Presynaptic Vesicular Release Machinery

**Figure 1:**
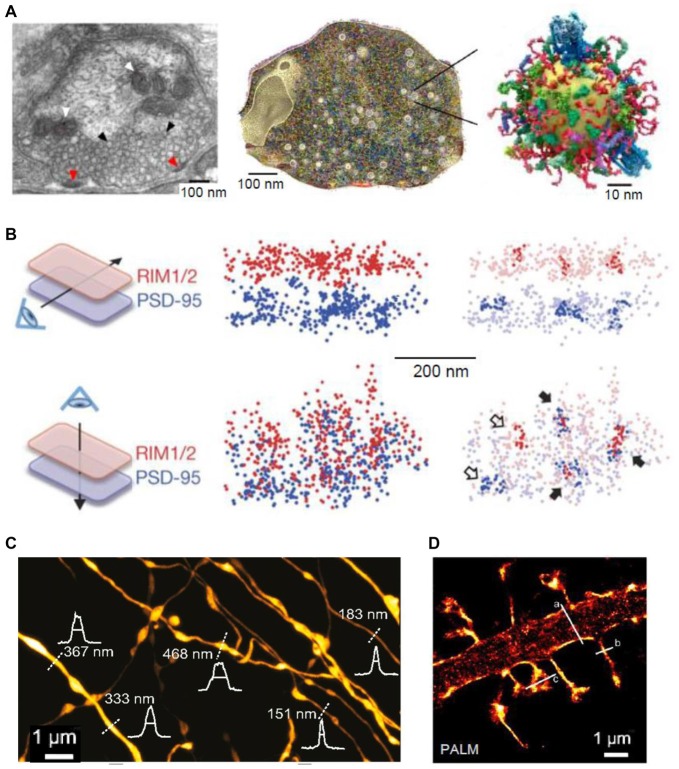
Super-resolution (SR) imaging of pre- and postsynaptic connections. **(A)** *Left*: electron micrograph depicting the ultrastructure of a presynaptic bouton; mitochondria (white arrowheads) appear positioned near the synaptic vesicles (black arrowheads). Fusion of synaptic vesicles takes place at the active zone (red arrowheads). *Middle*: three-dimensional (3D) reconstruction of the presynapse considering the copy numbers and localization of 60 synaptic proteins. *Right*: illustration of one synaptic vesicle; modified from Maidorn et al. (2016) with permission. **(B)** Trans-synaptic nanoscale alignment of active zone and postsynaptic density (PSD) proteins. Distributions of synaptic RIM1/2 and PSD-95 pair as the original localizations (*left*) and with nanoclusters highlighted (*right*); filled arrows, aligned nanoclusters; open arrows, non-aligned nanoclusters; modified from Tang et al. (2016) with permission. **(C)** Stimulated-emission depletion (STED) image of axons in the *stratum radiatum* in the CA1 area. Intensity profiles (white) and FWHM measurements (straight lines) of axon and bouton diameters were taken at the locations indicated by the white dotted lines; modified from Chéreau et al. (2017) with permission. **(D)** Photo-activated localization microscopy (PALM) of dendritic spines, acquired with a frame rate of 50 ms (8000 frames); modified from Izeddin et al. (2011) with permission.

The presynaptic specialization, or the “presynapse”, is a crowded yet highly dynamic axonal compartment, in which the finely tuned regulation of molecular interactions is essential for the function and integrity of synapses. Some key aspects pertinent to the molecular organization of the presynapse have been revealed using EM and mass spectrometry (Südhof, 2006; Takamori et al., 2006) and, more recently, with STED (Figure 1A; Wilhelm et al., 2014). The essential functions of presynapses include vesicular release and recycling of neurotransmitters, of which some critical aspects remain under debate. Clearly, single molecule imaging techniques could provide important clues in this regard (Rizzoli, 2014; Maidorn et al., 2016).

Various SR techniques have been used to image synaptic vesicles, their molecular composition and recycling mechanisms (Willig et al., 2006; Westphal et al., 2008; Kamin et al., 2010; Lehmann et al., 2015). STED imaging has revealed that synaptotagmin molecules remain clustered in isolated presynaptic membrane patches after synaptic vesicle fusion, suggesting that at least some vesicle constituents remain together during recycling (Willig et al., 2006). Another application of STED imaging showed that the plasma membrane SNAREs form clusters of ~60 nm diameter containing about 75 densely packed molecules in PC12 cells (Sieber et al., 2007; Maglione and Sigrist, 2013). FRAP experiments detected freely diffusing molecules that dynamically move between such clusters (Sieber et al., 2007). Direct STORM (dSTORM) imaging showed that, indeed, unclustered syntaxin as well as SNAP-25 molecules reside next to these clusters in PC12 cells (Bar-On et al., 2012). Furthermore, it has been shown that subsequent stimulation leads to endocytosis of a “readily retrievable” pool of synaptic vesicles (Hua et al., 2011). Assemblies of synaptotagmin molecules in the plasma membrane that might control membrane integrity or serve as molecular platforms for the formation of SNARE complexes have been identified in recent studies with STED (Milovanovic and Jahn, 2015; Maidorn et al., 2016). STED was also used to visualize, for the first time, vesicular trafficking on the nanoscale in real time (Westphal et al., 2008). The buffer zone for endocytosis was also imaged in a study combining confocal and STED microscopy, mechanical force measurements, pharmacology and gene knockout to investigate the dynamic assembly of filamentous actin that mediates Ω-profile vesicle merging at synapses (Wen et al., 2016). Additionally, actin dynamics were found to modulate presynaptic structure and function by dynamically controlling the active zone organization following local neuronal activity (Glebov et al., 2017). A recent study detected individual vesicle fusion events with ~27 nm localization precision at single hippocampal synapses under physiological conditions (Maschi and Klyachko, 2017). The researchers found that multiple distinct release sites could exist within individual synapses. These sites were distributed throughout the active zone and underwent repeated reuse in a dynamic and activity-dependent manner (Maschi and Klyachko, 2017). A recent study has documented trans-synaptic nanocolumns that regulate the positioning of pre- and postsynaptic scaffolding proteins (Figure 1B; Tang et al., 2016). Therefore, one possible explanation for the distinct release sites reported recently (Maschi and Klyachko, 2017) are the nanoclusters of presynaptic proteins (especially RIM1/2) at the active zone (Tang et al., 2016). Hence, RIM1/2 clusters could be seen as markers for release sites (Tang et al., 2016).

The notion that individual presynaptic proteins form nanoclusters regulating the aforementioned nanocolumns is further supported by studies using EM and STED. Such studies reveal the clustering of voltage-gated Ca^2+^ channels at synapses of auditory hair cells (Frank et al., 2010) and at the neuromuscular junction in adult and aged mice (Nishimune et al., 2016), by bassoon. Moreover, a recent STED-based study found that the presynaptic clustering of active zone proteins RIM-BP2 was required for the clustering of calcium channels and bassoon at hippocampal CA3-CA1 synapses (Grauel et al., 2016).

In addition to revealing the nanoclustering of active zone organizers, SR microscopy has been employed to confirm the presence of brain-derived neurotrophic factor (BDNF) in small granules in the presynaptic face of excitatory synapses in cultured neurons, allowing for a regulated anterograde release from glutamatergic synapses (Andreska et al., 2014). Moreover, STORM imaging of hippocampal slices demonstrated that Δ^9^-tetrahydrocannabinol (THC) treatment induces internalization and disappearance of type 1 cannabinoid (CB_1_) receptors from γ-aminobutyric acid (GABA)-ergic axon terminals (Dudok et al., 2015). A combination of whole-cell recordings and STORM was employed to investigate the role of local protein synthesis in rodent hippocampal slices. They found that presynaptic protein synthesis is required for long-term, but not short-term, plasticity of GABA release from CB_1_-expressing axons and might play a general role in controlling long-term plasticity in the mature mammalian brain (Younts et al., 2016). Another recent study investigated the distribution of dopamine transporters in cultured dopaminergic neurons using PALM and STORM (Rahbek-Clemmensen et al., 2017). The authors have found that the transporters cluster in multiple irregular nanodomains and that the clustering is dependent on cholesterol. Furthermore, the transporter nanodomains are non-overlapping with the loci of tyrosine hydroxylase and vesicular monoamine transporter 2 in potential presynaptic transmitter release sites (Rahbek-Clemmensen et al., 2017). In addition to imaging individual molecules in presynaptic sites, STED has been employed to visualize the structure and plasticity of axon shafts and boutons in mice hippocampal sections (Figure 1C; Chéreau et al., 2017).

## SR Imaging of the Postsynaptic Site

Both STED and SMLM have been implemented to analyze the structure of dendritic spines and the post-synapse. For example, in cultured neurons, STORM revealed the dynamics of thin filopodia and dendritic spines as well as of the endoplasmic reticulum and mitochondria (Shim et al., 2012). Additionally, STED has also been used to image the fine structure of dendritic spines (Nägerl et al., 2008; Ding et al., 2009; Tønnesen et al., 2011, 2014; Berning et al., 2012; Bethge et al., 2013). In addition to revealing macroscopic changes of dendrites and spines, STED (Urban et al., 2011; D’Este et al., 2015; Sidenstein et al., 2016) and SMLM techniques (such as sptPALM) have been used to investigate actin filaments and their dynamics in spines and dendrites and their role in spine maturation and function (Figure 1D; Tatavarty et al., 2009, 2012; Frost et al., 2010; Izeddin et al., 2011; Chazeau et al., 2014; MacGillavry et al., 2016; Wang et al., 2016). It has been found that dendritic actin polymerization is more complex and heterogeneous than previously thought, with different actin flows and random motions (Tatavarty et al., 2009; Frost et al., 2010). Moreover, enhanced actin polymerization was revealed in subdomains of the dendritic spine including the postsynaptic density (PSD) and the spine neck, which might play a role in the repositioning of glutamate receptors (Frost et al., 2010). Furthermore, the nucleation of actin extension seemed to start from a single point within the PSD, raising the possibility that changes in the environment are sensed by the actin filaments and in turn are translated into the PSD to mediate spine maturation (Izeddin et al., 2011; Chazeau et al., 2014). Among the organizing proteins of mature spine structure is synaptopodin, which exerts an indirect effect, via F-actin, on the diffusion of membrane proteins in the spine neck (Wang et al., 2016). The lattice-like structures of filamentous actin were also imaged *in vivo*, revealing a structure similar to that seen *in vitro* (Berning et al., 2012; Willig et al., 2014). A recent study used a far-red emitting fluorescent protein, mNeptune2, as a STED probe and achieved to resolve actin filaments in the living brain at a resolution of ~80 nm (Blazquez-Llorca et al., 2015). The researchers did not find any phototoxicity, confirming the advantage of far-red light for *in vivo* applications (Blazquez-Llorca et al., 2015). Altogether, SR techniques have substantially extended our view of the organization of the spine cytoskeleton revealing a much more crowded and organized cell compartment than previously thought (MacGillavry and Hoogenraad, 2015; Chazeau and Giannone, 2016).

In addition to the macroscopic structure of spines and the dynamics of their cytoskeleton, SR techniques have helped to decipher the nanoscopic organization of the PSD. The first study investigating the nanoscale structure of excitatory synapses was performed in fixed rat brain tissue (Dani et al., 2010). The group studied the nanoscopic distribution of pre- and postsynaptic scaffolding proteins and established a detailed 3D map of the synapses. Furthermore, they demonstrated variations in the organization of N-methyl-D-aspartate receptors (NMDARs) and α-amino-3-hydroxy-5-methyl-4-isoxazolepropionic acid receptors (AMPARs) across the main olfactory bulb (MOB) and the accessory OB (AOB; Dani et al., 2010). The researchers found an increased ratio of the NMDAR subunit NR2B to the AMPAR subunit GluR1 in the AOB, possibly representing a larger number of “immature” synapses and hence suggesting the potential for significant activity-dependent plasticity in the adult AOB. The authors confirmed this hypothesis using optogenetics to specifically mimic vomeronasal organ (VMO) activation, also detecting significant synapse maturation (an increase in the synaptic content of GluR1 and Homer1 and a moderate decrease in NR2B) upon light stimulation (Dani et al., 2010). The activity of AMPARs is key to memory formation and synaptic plasticity, and their insertion into and removal from synapses are tightly regulated (Newpher and Ehlers, 2008; MacGillavry et al., 2011). In 2013, three independent groups used different SR methods to discover that at excitatory synapses AMPARs accumulate into nanoscopic clusters (Fukata et al., 2013; MacGillavry et al., 2013; Nair et al., 2013). One group used PALM of several postsynaptic scaffolding molecules such as PSD95 and Homer1, as well as dSTORM of AMPARs and NMDARs in live and fixed neuronal cultures (MacGillavry et al., 2013). Another group employed a combination of techniques in live cultures investigating the dynamics of palmitoylated PSD95 (Fukata et al., 2013). The researchers used FRAP and PALM and found that the PSD was organized through subdomains of PSD95, which in turn are regulated through DHHC2-dependent palmitoylation (Fukata et al., 2013). Finally, uPAINT, sptPALM, dSTORM and STED were used to discover that AMPARs are immobile at nanodomains but diffuse freely between them, which could be due to the nanoorganization of PSD95 scaffolds (Nair et al., 2013). All the groups have concluded that AMPARs are organized at synapses in nanoclusters that are much smaller than the PSD (0–4 AMPAR nanodomains per PSD, with an average size of ~80 nm; Compans et al., 2016). Furthermore, the number of the AMPAR nanodomains depended on the size of the underlying PSD. The average center-to-center distance between the nanodomains is 500 nm suggesting that a single glutamate release cannot act on more than one domain (Nair et al., 2013). In these studies, dSTORM data revealed that 20–25 AMPARs are located in each nanodomain.

The organization of PSD95 molecules within the PSD was investigated further. The results revealed that, even though PSD95 can be found throughout the PSD, this protein, too, clusters in nanodomains of ~150 nm in diameter (Fukata et al., 2013; MacGillavry et al., 2013; Nair et al., 2013). These PSD95 nanodomains were also found in brain slices (Broadhead et al., 2016; Tang et al., 2016). Using a single transmembrane domain with a PDZ binding motif, the impact of the tight protein packing within the PSD was investigated leading to a conclusion that both crowding and binding dynamics limit diffusion and escape of AMPARs from the synapse (Li et al., 2016). Blocking the interaction of AMPARs with stargazin, which regulates AMPAR trafficking and gating, increased the rate of AMPAR diffusion as measured with sptPALM but revealed an interaction of the receptors with other proteins outside of the PSD (Hoze et al., 2012). Even though the diffusion properties of neuronal receptors had been investigated already in live cells using nanoparticle-based SPT, these novel studies were the first to describe the nanoorganization of AMPARs and of scaffolding proteins at PSDs.

The nanoorganization and its dynamic rearrangement opposing similar nanostructures of presynaptic priming and docking factors such as RIM1/2 was later confirmed in the aforementioned study by Tang et al. (2016). A similar alignment was seen at the neuromuscular junction in mice (York and Zheng, 2017). Using SIM and STORM, the authors located acetylcholine receptors to the edges of crests surrounding the opening of folds, therefore displaying a trans-synaptic alignment with the presynaptic active zones and enabling effective synaptic transmission (York and Zheng, 2017).

STED and SMLM have also been applied to assess the synaptic localization and the dynamics of other receptors, channels and scaffolding proteins. For example, STORM revealed that the A-kinase-anchoring protein 79/150 prompts the clustering of different ion channels (M-type K^+^, L-type Ca^2+^ and TRPV1) into functional domains, together with G protein-coupled receptors (Zhang et al., 2016). STED was used to analyze the size distribution, the association into nanoclusters and the long-range interactions of nicotinic acetyl-choline receptor nanoclusters and their cholesterol dependence (Kellner et al., 2007). The number and the nanoorganization of the Na^+^K^+^-ATPase at excitatory synapses, as well as clustering and colocalization with dopamine D1 receptor and DARPP-32, were assessed using STED and PALM (Blom et al., 2011, 2012, 2013, 2016). It has been found that the P2X7 receptor is stabilized in nanoclusters of ~100 nm diameter and that its trafficking shows confinement in synaptic areas (Shrivastava et al., 2013). Moreover, STED and sptPALM were employed to study the surface dynamics of calcium channel subunits in cultured cells (Voigt et al., 2016). A novel monomeric streptavidin dSTORM approach was employed to investigate the relative distribution of presynaptic neurexins (Nrx) 1β and its postsynaptic binding partners neuroligin (Nlg) 1 and LRRTM2 in primary hippocampal neurons (Chamma et al., 2016a,b).

The sub-synaptic distribution of molecular components in inhibitory (GABA-ergic) synapses has also been visualized using SR imaging techniques. For instance, quantitative 3D-PALM analysis has revealed the gephyrin organization in inhibitory synapses in cultured spinal cord neurons: the ensuring calculations estimated the postsynaptic clusters of 40–500 gephyrin molecules, packed at a density of about 5000 molecules/μm^2^, whereas synapses *in situ* contained about three times as many molecules and were packed more densely (Specht et al., 2013). The same group recently used quantitative PALM to show that alpha-1 and alpha-3 containing glycine receptors display the same α_3_:β_2_ stoichiometry and gephyrin binding (Patrizio et al., 2017). However, the authors found that alpha-1 containing receptors are more mobile and less densely packed (1100 molecules/μm^2^ vs. 1500 molecules/μm^2^; alpha-3 containing receptors) and that their number at synapses was reduced in response to interleukin 1β. This highlights an alpha subunit-specific mechanism of receptor-gephyrin binding as well as a cell-type dependent regulation of glycinergic currents (Patrizio et al., 2017). The nanoorganization of the postsynaptic scaffolding proteins gephyrin and PSD95 in cultured hippocampal neurons was suggested using STED imaging (Dzyubenko et al., 2016). The researchers counted nanodomains of PSD95 and gephyrin and found that their number is significantly increased when the postsynaptic puncta are paired with presynaptic puncta compared to unpaired clusters (Dzyubenko et al., 2016). Another elegant study combined STORM with array tomography to reconstruct the entire inhibitory synaptic input field of individual retinal ganglion and amacrine cells in mice and determined the spatial distribution and neurotransmitter receptor identity of the synapses therein (Sigal et al., 2015). Whilst such SR approaches cannot achieve spatial resolution of EM, they could reveal the 3D distribution of individual protein molecules on a larger, contiguous scale, the task unattainable by EM techniques.

## Imaging of Astrocytes and the Tripartite Synapse Using SR Methods

**Figure 2:**
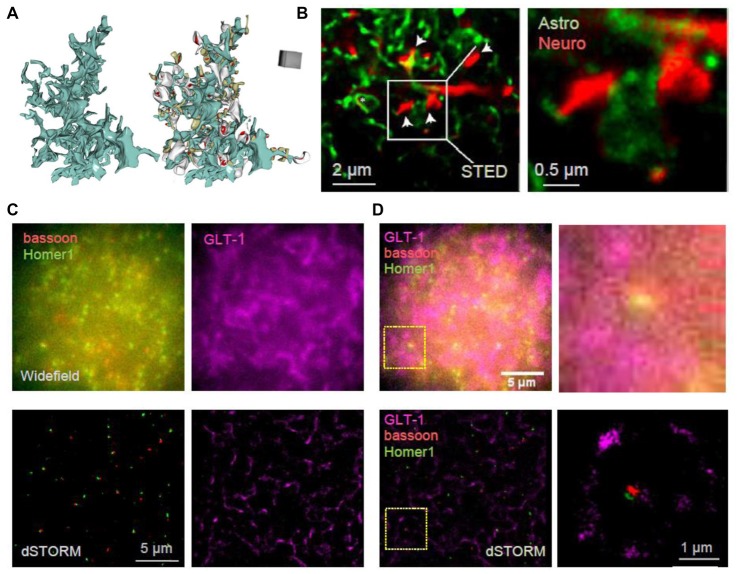
Towards deciphering the nanoscale architecture of tripartite synapses. **(A)** 3D reconstruction of an astrocyte fragment (blue) shown with and without adjacent thin (gray) and mushroom (dark yellow) dendritic spines equipped with PSDs (red); adapted from Medvedev et al. (2014) with permission. **(B)** STED images showing CA1 *stratum radiatum* astrocytic processes (green) adjacent to synaptic structures in organotypic slices (red; dendritic spines, arrows) at lower (*left*) and higher (*right*) magnification; square (*left*), magnified area; modified from Panatier et al. (2014) with permission. **(C)** In-house example: widefield (*top*) and direct STORM (dSTORM; *bottom*) images of bassoon (CF568-tagged, red), Homer1 (Atto488-tagged, green) and GLT-1 (Alexa647-tagged, magenta) of a 20 × 20 μm ROI in hippocampal area CA1; 40 μm thick hippocampal sections (500 g male rat); photoswitching buffer containing 100 mM cysteamine and oxygen scavengers (glucose oxidase and catalase; Metcalf et al., 2013); recorded with a Vutara 350 microscope (Bruker Corp., Billerica, MA, USA). **(D)** Same example as in **(C)** but shown as combined three-color widefield (*top*) and dSTORM (*bottom*) images, at lower (*left*) and higher (*right*) magnification; dotted squares (*left*), magnified area.

The visualization of live astrocytic processes and their signaling protein complexes has been technically challenging, mainly due to their nanoscopic size and the fact that their spatial separation could also be nanoscopic (Rusakov, 2015), as evidenced by the visualization of 3D-reconstructed images of perisynaptic astroglia (Figure 2A). Therefore, even though substantial advances have been made in the imaging of synaptic molecules, little is known about the molecular nanostructure of synaptic microenvironment associated with astroglia. Astrocyte cultures have been used to visualize cytoskeletal and receptor localization (reviewed in Heller and Rusakov, 2015) and to correlate subcellular cytoskeletal organization with cell morphology and membrane stiffness (Curry et al., 2017). The localization of the endoplasmic reticulum and mitochondria in live cultured cortical astrocytes has been assessed using SIM revealing their dynamic arrangements close to the basal plasma membrane, across the whole cultured cell (Brunstein et al., 2013). Implementing PALM and dSTORM has revealed the clustering behavior of AQP4 and Kir4.1 in cultured cells as well as in brain sections (Rossi et al., 2012; Smith A. J. et al., 2014; Smith and Verkman, 2015). Some ubiquitous astrocytic metabolic proteins, such as glutamine synthetase and S100β, have been used to reveal the nanoscopic structure of thin astroglial processes in fixed cultured cells and brain sections with STED and dSTORM (Volterra et al., 2014; Heller et al., 2017). Similarly, genetically-encoded fluorescent proteins that fill whole cells were used to dissect tripartite synapses with the help of STED (Figure 2B; Panatier et al., 2014). Another approach achieved near SR resolution using the Zeiss Airyscan method to reveal thin astrocytic processes in cleared tissue (Miller and Rothstein, 2016). A combination of EM and STED confirmed the presence of astrocytic and NG2 cell processes at nodes of Ranvier (Serwanski et al., 2017).

Intriguingly, two recent studies asked whether SR microscopy was technically feasible or advantageous when applied to cryo-cut human brain tissue. The first study revealed serotonin receptor clusters using a dSTORM approach on neuronal and astrocytic elements (Jacak et al., 2015). The second study compared STORM and SR optical fluctuation imaging (SOFI) as means to reveal axons and astrocytic processes (Hainsworth et al., 2017). It was found that STORM underestimated the width of axons and astrocytic processes and even though SOFI has lower spatial resolution it provided more accurate width measurements (Hainsworth et al., 2017). A recent elegant study investigated local translation in astrocytic processes (Sakers et al., 2017) using STORM to illuminate ribosomes in the close proximity of synapses where the locally produced proteins may alter synapse plasticity.

Imaging the tripartite synapses in fixed brain sections using dSTORM has also been progressing. Three-color 3D dSTORM could reveal the positioning of glial glutamate transporter GLT-1 around excitatory synapses delineated by the tight pre- and postsynaptic clusters of bassoon and Homer1, respectively (Figures 2C,D). In addition to imaging vesicle dynamics in presynaptic structures, SR methods have been used to assess the merging of vesicles with the plasma membrane in astrocytes, revealing similar release mechanisms (Singh et al., 2014; Li et al., 2015; Guček et al., 2016; Jorgačevski et al., 2017). Recently, SIM and high-resolution cell-attached capacitance measurements have been used to show that dynamin regulates fusion pore geometry and kinetics of endo- and exocytotic vesicles in cultured astrocytes (Lasic et al., 2017).

## Combining Functional Synaptic Studies with SR Imaging

Clearly, electrophysiological or optical readout of synaptic function is essential for achieving reliable interpretation of SMLM data pertinent to the molecular dynamics of synaptic (and extra-synaptic) components (Hosy et al., 2014; Compans et al., 2016). One of the earliest several recent studies have assessed dynamic changes of receptor clustering upon synaptic stimulation. Following induction of long-term potentiation (LTP) of inhibitory transmission (iLTP), gephyrin was found to accumulate at inhibitory synapses leading to an increased number of synaptic GABA_A_ receptors (Petrini et al., 2014; Pennacchietti et al., 2017). The authors found that the nanoscale organization of inhibitory synaptic proteins determined inhibitory synaptic plasticity, thus adding a further level of complexity to the regulation of the neuronal network activity by plastic inhibitory synaptic signals (Pennacchietti et al., 2017). PALM and STORM were used to investigate the trafficking of CamKII in spines and revealed that the protein exists in three kinetic states, slow (interaction with immobile substrates), intermediate (binding to actin), and fast (CamKII alone), whereas NMDAR activation slowed down diffusion rates (Lu et al., 2014). Furthermore, it was found that the phosphorylation of AMPARs by CamKII was crucial for P2X2-mediated AMPAR internalization and ATP-driven synaptic depression (Pougnet et al., 2014, 2016).

**Figure 3:**
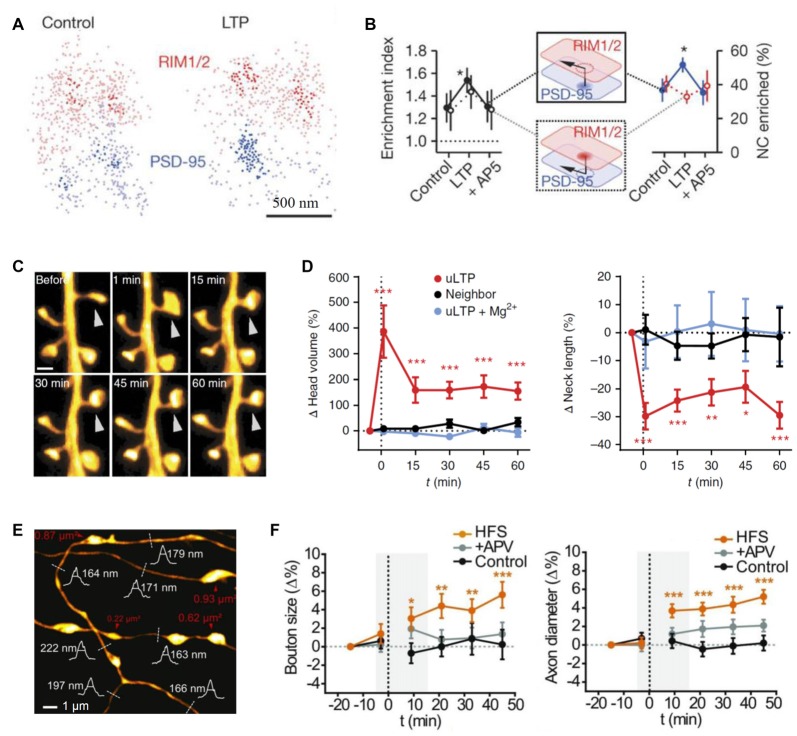
Probing synaptic plasticity on the nanoscale. **(A)** Space distributions of synaptic RIM1/2 and PSD-95 proteins, in control and post-long-term potentiation (LTP) conditions, with nanoclusters highlighted. **(B)** Across-condition comparison of enrichment index and the percentage of nanoclusters enriched (*n* = 45, 87 and 42 synapses for control, LTP and AP5, respectively); modified from Tang et al. (2016) with permission. **(C)** Monitoring dendritic spine morphology following glutamate uncaging-induced LTP (uLTP) protocol; arrowhead, uncaging spot. **(D)** The dynamics of spine head size (*left*) and the neck length (*right*) following uLTP protocol; modified from Tønnesen et al. (2014) with permission. **(E)** An example of stimulated-emission depletion (STED) imaging employed to monitor long-term dynamics of axonal morphology following high frequency stimulation (HFS, a protocol to induce LTP). **(F)** Time source of the (normalized) axonal bouton size (*left*) and axon diameter (*right*) in three conditions: control, HFS and APV (N-methyl-D-aspartate receptors, NMDA receptor blockade); dotted line, HFS onset; gray shadows, time window of short-term observations (as in Figure 2B); modified from Chéreau et al. (2017) with permission. *p < 0.5, **p < 0.1, ***p < 0.01.

SR was used to discover that ankyrin-G accumulates in dendritic spines after induction of chemical LTP and that its knockdown prevents spine head enlargement (Smith K. R. et al., 2014). Chemical LTP also induces a matrix metalloproteinase-dependent enlargement of a subset of small spines and the immobilization, synaptic accumulation and clustering of AMPARs at dendritic spines (Szepesi et al., 2014). Interestingly, the induction of long-term depression (LTD) led to an increased nanodomain size of the cell adhesion molecule SynCAM 1 (Perez De Arce et al., 2015), and ephrin B3 was found to regulate the localization of PSD95 to stable synapses and in stable nanodomains (Hruska et al., 2015). Interestingly, presynaptic RIM1/2 molecules cluster in a similar way to postsynaptic PSD95 molecules, and both nanodomains co-align across the synaptic cleft (Figure 1B; Tang et al., 2016). Furthermore, after LTP induction these nanocolumns remain, with an increase in PSD95 clusters (Figures 3A,B), thus providing further evidence for the trans-synaptic organization of scaffolding molecules. The mechanisms that mediate this clustering are yet to be discovered. A recent elegant study combined PALM and optogenetics to control the insertion of PSD95 molecules and hence AMPARs into established PSDs (Sinnen et al., 2017). It appears that simple insertion of the receptors was not enough to drive synaptic potentiation. Therefore, more complex changes in the nanodomain composition and characteristics are required for spine maturation and the gating of synaptic strength. STED has been used mainly to visualize the morphology of neuronal structures and how they change upon plasticity. For example, STED revealed an increased spine head volume and a decreased spine neck length after LTP induction (Figures 3C,D; Tønnesen et al., 2014). Furthermore, STED imaging has been used to investigate morphological plasticity of synaptic boutons and axon shafts which dynamically fine-tune action potential conduction velocity (Figures 1C, 3E,F; Chéreau et al., 2017). Another study used a combination of AMPAR immobilization approaches together with FRAP and uPAINT to evaluate the impact of AMPAR surface diffusion on hippocampal LTP and contextual learning (Penn et al., 2017). The authors have shown, in brain slices and *in vivo*, that AMPAR lateral diffusion and synaptic trapping is an integral part of the early phases of hippocampal-dependent fear learning (Penn et al., 2017).

It appears that only one study to date has used SR microscopy to investigate astrocyte dynamic during plasticity conditions. The researchers evaluated the role of BDNF as a gliotransmitter *in vivo* using EM and SIM in combination with LTP-induction (Vignoli et al., 2016). It was found that BDNF recycling by glial cells was required for memory consolidation, the latter being followed by highly localized TrkB phosphorylation on adjacent neurons (Vignoli et al., 2016).

## Concluding Remarks

There have been a growing number of elegant studies that employed SR technology to shed light on the nanoscopic events pertinent to synaptic function and plasticity. However, the majority of them have been conducted either in cultured cells or in fixed brain sections. Whilst these data have moved the field forward, the goal post is now to achieve SR imaging in organized, preferably live, brain tissue. This is particularly important for astrocytes whose morphology in culture is drastically different from that *in situ*.

In this respect, STED imaging has been ahead of the field providing live imaging *in situ* (Urban et al., 2011; Berning et al., 2012; Gao et al., 2012; Chen et al., 2014; Panatier et al., 2014; Chéreau et al., 2017) including intact animals (Berning et al., 2012; Willig et al., 2014; Blazquez-Llorca et al., 2015). Clearly, achieving reliable SR results *in situ* faces technical challenges, such as restricted optical access, sample movement, or optical aberrations induced by the specimen. In addition, organized brain tissue is packed with nanoscopic cellular compartments of neuronal or astroglial origin that have to be optically separated from the structures of interest. One possible way towards overcoming these difficulties is adaptive optics (Booth et al., 2015), which improves the signal-to-noise ratio, axial resolution, and depth penetration as shown using *in vivo* two-photon excitation imaging (Rueckel et al., 2006; Ji et al., 2012; Wang et al., 2014) or STED (Gould et al., 2012; Patton et al., 2016). In parallel, further improvements in performing multi-color imaging are needed, which depends on the progress in achieving the required spectral properties of fluorescent labels and the optics equipment and analyses involved. So far only large cellular structures such as dendritic spines and their actin dynamics at a resolution of just under 100 nm have been visualized in the living brain (Berning et al., 2012; Willig et al., 2014; Blazquez-Llorca et al., 2015). Nevertheless, advances in reducing phototoxicity have enabled longer-term imaging. One common drawback of these approaches is that it is difficult to label endogenous proteins *in situ*, due to the limited access, which prompted the use of overexpressed proteins.

At present, 3D SR microscopy can be employed efficiently in many laboratories using custom build or commercial microscopes helping to reveal structures that are beyond the diffraction limit of light (Huang et al., 2008; Juette et al., 2008; Heller et al., 2017). The development and adaption of novel SR techniques such as qPAINT, which permits quantitative SR imaging in cellular structures (Jungmann et al., 2016), integrating exchangeable single-molecule localization (IRIS; Kiuchi et al., 2015) and 4Pi single-molecule switching nanoscopy, which allows volumetric reconstruction with 10- to 20-nm isotropic resolution of ~10-μm-thick samples (Huang et al., 2016), should increase the available resolution even further. The development of novel labeling techniques and probes is an important factor in improving resolution. For example, the use of photoactivatable, genetically encoded calcium indicators (Berlin et al., 2015) opens a door into nanoscopic calcium events in the cells studied. Similarly, the use of smaller labeling probes such as nanobodies (Pleiner et al., 2015) and aptamers (de Castro et al., 2016), monomeric streptavidin (Chamma et al., 2017), or the pore-forming bacterial toxin streptolysin O to label intracellular proteins in living mammalian cells for SR microscopy (Teng et al., 2016, 2017) should accelerate this progress. In parallel, the recent invention of other methods such as expansion microscopy (Chen et al., 2015; Chozinski et al., 2016; Tillberg et al., 2016) should make SR probing more affordable for many laboratories. Finally, SR imaging has already been applied to investigate established models of neurological disease, such as Alzheimer’s (Šišková et al., 2014; Schedin-Weiss et al., 2016), intellectual disabilities (Wijetunge et al., 2014; Barnes et al., 2015), amyotrophic lateral sclerosis, and frontotemporal dementia (Schoen et al., 2016). The new discoveries in SR should certainly help to translate these findings into the quest for the efficient therapeutic intervention.

The continued emergence of SR techniques has been opening new horizons in our exploration of the perisynaptic nano-world. Whilst the traditional EM methods have been able to reveal the fine architectural features of synaptic coverage by PAPs, SR can provide us with unique tools to answer some fundamental questions pertinent to the synaptic machinery and its microenvironment. How are the key signaling molecules (neurotransmitter transporters, potassium channels, aquaporins, ion exchangers, etc.) distributed in neuronal and astroglial membranes outside synapses? Do PAPs and their protein complexes re-arrange upon neural activity and/or the induction of synaptic plasticity? From what perisynaptic astroglial loci are gliotransmitters released? Where do mitochondria and internal calcium stores, if any, occur in PAPs? Does the synaptic nano-architecture reflect functional identities of individual synapses? Answering these and related questions should give us a previously unattainable quantitative basis to decipher molecular mechanisms acting at and around synapses, at the level of biophysical interactions among individual molecules.

## Author Contributions

JPH and DAR wrote the manuscript.

## Conflict of Interest Statement

The authors declare that the research was conducted in the absence of any commercial or financial relationships that could be construed as a potential conflict of interest.
